# Green extraction of puromycin-based antibiotics from *Streptomyces albofaciens* (MS38) for sustainable biopharmaceutical applications

**DOI:** 10.3389/fchem.2023.1326328

**Published:** 2024-01-09

**Authors:** Neha Singh, Sandip Patil, Mohd. Shahnawaz, Vibhuti Rai, Abhinandan Patil, C. K. M. Tripathi, Feiqiu Wen, Shaowei Dong, Defeng Cai

**Affiliations:** ^1^ Biochemistry and Microbiology Laboratory, School of Studies in Life Sciences, Pt. Ravishankar Shukla University, Raipur, India; ^2^ Virology Lab, Department of Microbiology, Pandit Jawahar Lal Nehru Memorial Medical College, Raipur, Chhattisgarh, India; ^3^ Department of Haematology and Oncology, Shenzhen Children’s Hospital, Shenzhen, Guangdong, China; ^4^ Paediatric Research Institute, Shenzhen Children’s Hospital, Shenzhen, Guangdong, China; ^5^ Department of Botany, University of Ladakh, Ladakh UT, India; ^6^ Division of Pharmacy, Dr. DY Patil University, Kolhapur, Maharashtra, India; ^7^ Fermentation Technology Division, Central Drug Research Institute, CSIR, Lucknow, India; ^8^ Clinical Laboratory (Pathology) Centre, South China Hospital of Shenzhen University, Shenzhen, Guangdong, China

**Keywords:** *Streptomyces albofaciens* strain MS38, secondary metabolites, antimicrobial activity, green extract-antimicrobial compound, microbial biodiversity, structural elucidation

## Abstract

**Background:** Microbial secondary metabolites have shown promise as a source of novel antimicrobial agents. In this study, we aimed to isolate, characterize, and evaluate the antimicrobial activity of compound from a novel *Streptomyces albofaciens* strain MS38. The objective was to identify a potential bioactive compound with broad-spectrum antimicrobial properties.

**Methods:** The isolated strain MS38 on starch casein agar was characterized using morphological, physiological, and molecular identification techniques. The compound was obtained from the fermented broth through extraction with n-butanol and further purification using silica gel column chromatography and high-performance liquid chromatography (HPLC). Structural elucidation was conducted using Ultraviolet (UV), Infrared (IR), nuclear magnetic resonance (NMR), and mass spectrometry (MS) techniques. The antimicrobial activity was evaluated using the agar well diffusion method and the microplate Alamar blue assay (MABA).

**Results:** The isolated strain MS38 was identified as novel *S. albofaciens* based on morphological characteristics and confirmed by 16S sequences analysis and MALDI-TOF MS. The compound obtained from the fermented broth exhibited substantial antimicrobial activity against a variety of pathogenic bacteria and fungi. Structural analysis revealed a complex chemical structure with characteristic functional groups indicative of potential antimicrobial properties. The compound demonstrated strong activity against both Gram-positive (*Staphylococcus* Spp.) and Gram-negative (*Klebsiella pneumoniae* and *Escherichia coli*) bacteria, as well as fungi, including *Candida albicans* and *Trichophyton rubrum*.

**Conclusion:** This study successfully isolated and characterized a bioactive compound from a novel *S. albofaciens* MS38. The compound exhibited significant antimicrobial activity against a range of pathogenic microorganisms. These findings underscore the importance of exploring microbial biodiversity for the discovery of novel antimicrobial agents. This study contributes to the growing knowledge of microbial secondary metabolites with potential therapeutic value.

## 1 Introduction

In the ever-evolving landscape of antibiotic research, the quest for novel bioactive compounds with potent antimicrobial properties continues to be of paramount importance (Mantravadi et al., 2019). The emergence of drug-resistant pathogens poses a significant threat to global public health, underscoring the urgent need for innovative solutions (Serwecińska et al., 2020). In this pursuit, the present study unveils a promising avenue through the discovery of a novel strain, *Streptomyces albofaciens* (MS38), which demonstrates remarkable potential in producing puromycin-based antibiotics with efficacy against a wide array of pathogens. Antibiotics derived from natural sources have long served as a cornerstone in the battle against infectious diseases (Pancu et al., 2021). *Streptomyces* species, known for their prolific secondary metabolite production, have historically yielded a plethora of bioactive compounds (Alam et al., 2022). The isolation of *S. albofaciens* (MS38), coupled with its ability to synthesize puromycin-based antibiotics, marks a significant milestone in harnessing the therapeutic potential of microbial diversity. Puromycin, a well-known translation inhibitor, has exhibited promising antibacterial properties, particularly in targeting pathogenic bacteria with high translational activity (Aviner, 2020). This is naturally produced by *Streptomyces alboniger and* among the genus *Streptomycesis* as largest genus among the bacteria in terms of its potential to produce a wide range of antimicrobials used to treat human and animal diseases (Procópio et al., 2012; Caicedo-Montoya et al., 2021). Globally, >258 antibiotics play a critical role in combating diseases and safeguarding public health. Remarkably, ∼70 of these antibiotics recovered from a *Streptomyces* spp. (Procópio et al., 2012; WHO, 2021). *Streptomyces*, renowned for their prolific secondary metabolite production, has become an invaluable source of bioactive compounds, many of which have been harnessed for their potent antimicrobial properties (Gomez-Escribano et al., 2014). Building upon this foundation, the current study delves into soil isolate *S. albofaciens* (MS38), shedding light on the production of puromycin-based antibiotics from this isolate. Emergence and dissemination of antimicrobial resistance pose a threat to global public health (EClinical Medicine, 2021). Consequently, there is an imperative need for the discovery of innovative antibacterial interventions (Atanasov et al., 2021). The pursuit of novel pharmacologically active compounds from natural reservoirs has yielded a plethora of clinically significant therapeutics essential for addressing diverse human ailments (Atanasov et al., 2021). Particularly for conditions encompassing immunomodulation and infectious diseases caused by both bacteria and fungi, natural products continue to serve as a pivotal source for groundbreaking therapeutic agents (Wang et al., 2021). In the ongoing battle against antimicrobial resistance, the discovery of innovative antibacterial modules remains an urgent scientific and public health priority. The exploration of natural sources to uncover pharmacologically active agents has yielded invaluable therapeutic options crucial for managing a spectrum of human disorders. The novelty of *S. albofaciens* (MS38) lies not only in its capacity to produce puromycin-based antibiotics but also in the spectrum of pathogens these antibiotics effectively combat. The tested pathogens encompass a diverse array of strains, mirroring the complexity of challenges posed by multidrug-resistant microbes. The demonstrated efficacy of these antibiotics against such a wide range of pathogens holds immense promise for combating infections that have otherwise proven recalcitrant to traditional antibiotic treatments. Moreover, the current research delves into the optimization of fermentation conditions to enhance the yield of puromycin-based antibiotics. This critical aspect of the study addresses the practical translational potential of the findings, envisioning a future where the production of these bioactive compounds can be scaled up for therapeutic applications. The meticulous exploration of fermentation parameters not only optimizes yield but also highlights the potential for cost-effective production, a pivotal consideration in the journey from laboratory discovery to clinical implementation.

## 2 Materials and methods

### 2.1 Sample collection

A total of 125 samples were collected in sterile zip lock bags from a forested area near the Shivanth River, situated in Chhattisgarh, India (Coordinates: 21.190449 Latitude; 81.284920 Longitude). The protocol for collection was adapted from our prior studies (Singh et al., 2012; Singh N and Rai V, 2013). The soil samples underwent serial dilution up to 10-2 utilizing sterile saline water (0.85% NaCl). Subsequently, 100 µL of each diluted sample was spread on yeast malt extract (YME) agar medium within Petri dishes. These inoculated dishes were allowed to stand at room temperature for 10 min, facilitating liquid absorption, and were then incubated at 28°C at aerobic conditions for a duration of 7 days.

### 2.2 Isolation and characterization of MS38 isolate

Post-incubation, all grown *Streptomyces* bacterial isolates were subjected to morphological characterization, followed by 16S rRNA PCR assay using universal primers F-5′AGAGTTTGATCMTGGCTCAG3′ and R-5′ TACGGYTACCTTGTTACGACTT 3′, alongside MALDI-TOF MS. Genomic DNA extraction employed the Phenol-Chloroform Method, and DNA quality was assessed using a nanodrop. A260/A230 values between 2.0 and 2.2 were deemed suitable for subsequent PCR assays (Liu et al., 2022). The 16S rRNA PCR assay consisted of an initial denaturation at 94°C for 5 min, followed by 25 cycles encompassing denaturation at 96°C for 30 s, annealing at 58°C for 30 s, extension at 72°C for 1 min, and concluded with a final extension at 72°C for 5 min. Amplified PCR products were resolved using 1.2% agarose gel and visualized through ethidium bromide staining. PCR products were extracted using a Qiagen Extraction Kit and sequenced at the Institute of Microbial Technology (IMTECH), Chandigarh, India. Sequence analysis utilized the standard nucleotide-nucleotide BLAST program available at www.ncbi.nlm.nih.gov (last accessed on 00/00/0000). After BLAST analysis, a group of potential orthologs with over 98% sequence identity to the query sequences were selected for further scrutiny, with the omission of non-cultured and repetitive strain sequences. Phylogenetic analysis and nucleotide conservation were carried out using the neighbour-joining method via the CLUSTALW v1.83 web tool at http://www.ebi.ac.uk/clustalw (last accessed on 25/06/2022). Phylogenetic analysis of the 16S rRNA sequences of the isolates and closely related individuals was performed to gain insights into their evolutionary relationships. The Maximum Likelihood approach and Tamura-Nei model were employed to deduce the evolutionary history. The tree with the highest log probability (−3360.81) was displayed, with branch lengths scaled to substitutions per site. A dataset comprising 1877 locations was created, including codon positions 1st + 2nd + 3rd + Noncoding. Evolutionary analysis was conducted using MEGA11. Isolate MS38 was submitted to the National Culture Collection Centre, Institute of Microbial Technology, Chandigarh, India, and subsequently accessioned. The 16S rRNA gene sequence was also submitted to NCBI and accessioned accordingly.

### 2.3 Antimicrobial activity of the isolate MS38

Pathogenic and non-pathogenic test organisms were sourced from two institutions: the Microbial Type Culture Collection (MTCC, IMTECH, Chandigarh, India) and Jawaharlal Nehru Medical College (JNMC, Raipur, India) (Supplementary File S1). The antimicrobial activity was assessed against these test organisms. After cultivating the active isolate MS38 in the production medium of g/L glucose, soybean meal, sodium chloride, calcium carbonate, and pH 7.2 for 96 h at 180 rpm in a rotatory shaker (Remi Sales Engineering Ltd., Banglore), the antimicrobial activity of the fermented broth was evaluated using the cup-plate method (Balouiri et al., 2016). The test organisms were swabbed onto the surface of the proper bacterial solid medium-Muller Hinton agar and fungal solid medium- RPMI with 2% glucose, respectively, 100 µL of fermented broth was added into the well and incubated at the appropriate growth temperatures (37°C for bacteria and 28°C for fungus). The experiments were performed at least thrice for confirmation.

### 2.4 Fermentation

To optimize production of the bioactive compound, the isolate MS38 was cultivated in different production medium yeast-extract-malt-extract broth g/L (dextrose 10, peptone 5.0, malt extract 3.0, yeast extract 3.0), glucose soybean broth g/L (glucose 10, soybean meal 10, NaCl 10, CaCO3 1.0), peptone broth g/L (peptone 10, NaCl 5.0), starch casein nitrate broth g/L (soluble starch 10, casein 0.3, KNO3 2.0, MgSO4.7H2O. 0.05, K2HPO4 2.0, NaCl. 2.0, CaCO3 0.02, FeSO4.7H2O 0.01). All the 500 mL flasks containing 100 mL of production medium were inoculated with isolate MS38 and incubated at rotatory shaker at 180 rpm, 37°C for 16 days. Medium showing the highest antimicrobial activity in the form of zone of inhibition (mm) was selected. Bioactive compound production at different temperatures 15°C, 20°C, 25°C, 30°C, 37°C, 40°C as well as different rpm were 100 rpm, 120 rpm, 160 rpm, 200 rpm were also resolute. In the first stage, the active isolate MS38 was cultivated in 250 mL Erlenmeyer flasks, containing 50 mL of growth medium composed of yeast extract, malt extract, and glucose broth for seed culture preparation. This culture was incubated at 28°C and 180 rpm on a rotary shaker for 48 h. The second stage involved the transfer of 2% inoculum from seed culture in a 1 L conical flask with 200 mL of glucose-soybean meal broth medium containing glucose 10 g/L, soybean meal 10 g/L, NaCl 10 g/L, and CaCO3. The third stage included inoculation of the 2% seed inoculum from the 2^nd^ stage to ferment in a 3 L fermenter containing 1.5 L of glucose-soybean meal broth. The fermentation process was maintained at an aeration rate of 1 vvm and a pH of 7.5, with foam controlled using sterile silicone (Hi-media, India) for 3 days.

### 2.5 Extraction and purification of active compound

Following fermentation, the broth was centrifuged at 10,000 g for 20 min and then filtered through Whatman No. 1 filter paper. The clear filtrate was assessed for antibacterial efficacy against various test species. The antimicrobial compound was extracted from the filtrate using n-butanol in a 1:1 (v/v) ratio. The organic phase containing the active ingredient was concentrated to dryness using a rotary evaporator at 30°C and vacuum. Purification of the antibacterial agent was conducted using a silica gel column (4.2 × 70 cm). Sixteen purified fractions were obtained using a gradient of methanol and chloroform. Active fractions were pooled and further purified using a second silica column. The partially purified active metabolites were subjected to additional purification using HPLC (Chiu et al., 2016).

### 2.6 Investigation of the physico-chemical properties of the antimicrobial compound

The UV spectrum (200–500 nm) was measured using a PerkinElmer Lambda-25 UV spectrophotometer. IR spectra (200–4,000 cm^-1^) were recorded on an FT–IR PerkinElmer RX-1 spectrometer. The mass spectrum was recorded using a micromass Quattro II triple quadrupole mass spectrometer. 1H and 13C nuclear magnetic resonance (NMR) spectra were obtained using a 300 MHz Varian Inova instrument in deuterated water (D2O). Elemental analysis was performed using a Carlo Erba 1106 CHNO/S elemental analyzer. The melting point was determined using a WRS-1B digital melting-point apparatus (Singh et al., 2013).

### 2.7 Chemical reactions

Various chemical reactions were conducted, including Molish, Fehling, Sakaguchi, Ehrlich, Millon, and Mayer, and reactions with ninhydrin, nitroprusside, lead sulfate, and ferric chloride (Umezawa, 1977).

### 2.8 Biological activity

The minimum inhibitory concentration (MICs) of the purified antibacterial agent was determined using the serial dilution method according to the 2000 recommendation of the Clinical and Laboratory Standards Institute (CLSI) (CLSI, 2020). The microplate Alamar blue assay (MABA) was used to assess anti-mycobacterial activity. *M. tuberculosis* H37Ra and *M. tuberculosis* H37Rv suspensions were prepared at a concentration of approximately 105 cells per millilitre. The purified *S. albofaciens* MTCC 10793 compound (at concentrations of 10 g/mL) was added to the bacterial suspension, and the outcomes were assessed using the Alamar blue dye after 6 days of incubation.

## 3 Results

A total of 57 isolates were recovered from the soil sample among them five have antimicrobial potential and are considered for further studies. Out of these five, isolate MS38 was the best antimicrobial compound producing and chosen for further detailed studies.

### 3.1 Characterization of isolate MS38

**FIGURE 1:**
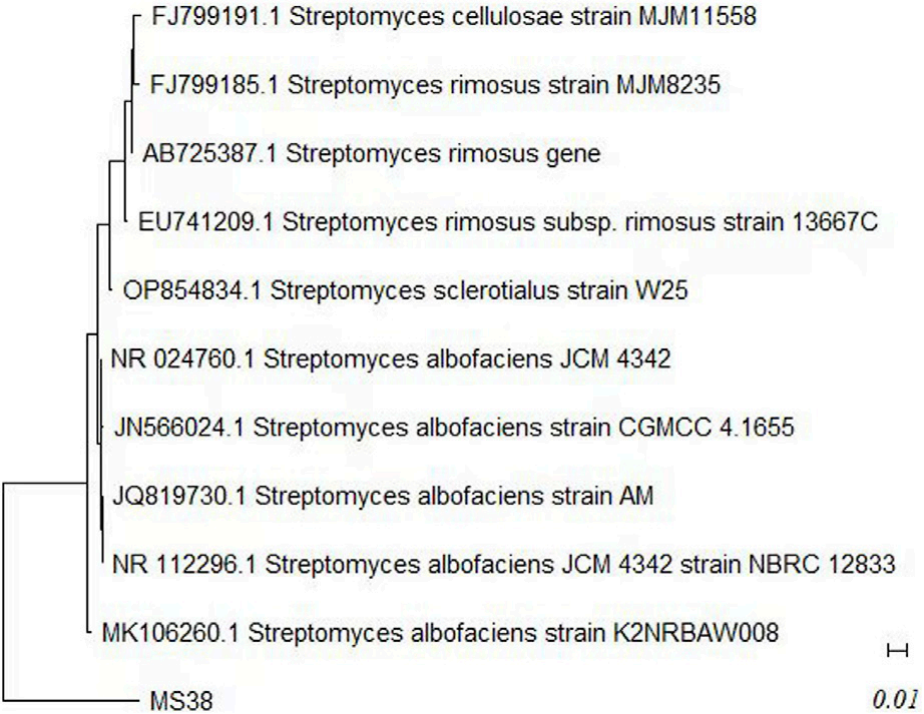
Phylogenetic tree of MS38 (*Streptomyces albofaciens* MTCC 10793) isolate based on MaximumLikelihood method and Tamura-Nei model using MEGA 11.

solate MS38 was tentatively assigned to the *Streptomyces* genus based on a comprehensive analysis of its morphological, physiological, and biochemical traits. Subsequent investigations involving 16S rRNA homology led to its definitive identification, resulting in the accession number MTCC 10793 and classification as *S. albofaciens*. The comparison of 16S rRNA partial sequences with the Basic Local Alignment Search Tool (BLAST) data in the NCBI database was facilitated using the MEGA 4.0.2 software, renowned for its evolutionary analysis capabilities (Figure 1). Further analysis, adhering to the criteria outlined by Bosshard et al. (2003) and relying on the generated dendrogram, affirmed that isolate MS38 was identical *to S. albofaciens* (GenBank accession no. AB045880) with a score of 98.63% sequence similarity in the 16S rRNA region. Consequently, isolate MS38 was confidently classified as *S. albofaciens* strain MS38 (Accessioned MTCC 10793).

### 3.2 Antimicrobial activity of isolate MS38

Isolate MS38, a local actinomycete strain, exhibited robust growth with a well-developed branching substrate and aerial mycelia, characterized by hyphal diameters ranging from approximately 0.3–0.6 μM. The spore chains displayed a distinctive spiral arrangement, and the mature spore mass exhibited a gray, moist appearance. The rugose surface of each spore, coupled with its diverse growth characteristics across all ISP mediums, contributed to its unique profile (Table 1).

**TABLE 1:** Cultural characteristics of *Streptomyces* albofaciens MS38.

Sr. No.	Media used	Growth	Front color	Reverse side color
1	Yeast extract-malt extract agar	Good Abundant	Gray	Brown
2	Inorganic salts- starch agar	Good Abundant	Gray	Yellowish brown
3	Oatmeal agar	Good Abundant	Gray	Light brown
4	Glycerol-asparagine agar	Moderate	White	Light brown
5	Tyrosine agar	Moderate	None	Yellowish brown
6	Peptone-yeast extract iron agar	Poor Moderate	White	Dark brown
7	Sucrose asparagine agar	Good	None	Light Brown
8	Sucrose nitrate agar	Good moderate	white and gray	Light brown

### 3.3 Physiological traits

The strain demonstrated optimal growth within a temperature range of 20°C–45°C and a NaCl concentration of 2–20 g/L. Notably, the strain exhibited utilization capabilities for various carbon sources, including D-glucose, D-xylose, L-arabinose, L-rhamnose, D-fructose, melibiose, melezitose, xylitol, maltose, lactose, mannitol, inositol, L-rhamnose, L-raffinose, sucrose, glycerol, D-ribose, sorbitol, galactose, L-cysteine, L-valine, L-leucine, L-asparagine, L-hydroxyproline, and L-serine. However, the utilization of D-cellubiose, meso-Dextran, L-phenylalanine, and L-histidine remained uncertain. Detailed TLC analysis of whole-cell hydrolysates revealed the presence of L, L-DAP, and asparagine in the cell wall composition, while notable absences included arabinose, galactose, glycine, and xylose (data not shown).

### 3.4 Antimicrobial compound production and extraction

The optimum production medium for the biosynthesis of the active metabolite by the isolate MS38 was recorded in glucose soybean meal medium at 37°C and 180 rpm with the zone of inhibition 36 mm against *Staphylococcus aureus* MTCC 96 and 28 mm against *Candida albicans* MTCC 1637. The production of antimicrobial compounds and biomass was monitored over 16 days (data not shown). Antibiotic synthesis was observed after 16 h of incubation, peaking during the stationary phase at 96 h and maintaining stability for approximately 168 h in shake flasks. Following the fermentation process, the resulting broth underwent filtration through a Buchner funnel with Whatman filter paper No. 1, followed by centrifugation for 20 min at 8,000–10,000 rpm and 28°C. Subsequently, the clear filtrates containing active metabolites underwent pH adjustment to 7.2, and the antibacterial molecule was extracted using n-butanol at a 1:1 (v/v) ratio. An organic phase was collected and subjected to evaporation under reduced pressure at 30°C using a rotavapor (Rotavapor R-300, Buchi, Mumbai, India), yielding a dark yellow powdered substance.

### 3.5 Purification and analysis

**FIGURE 2:**
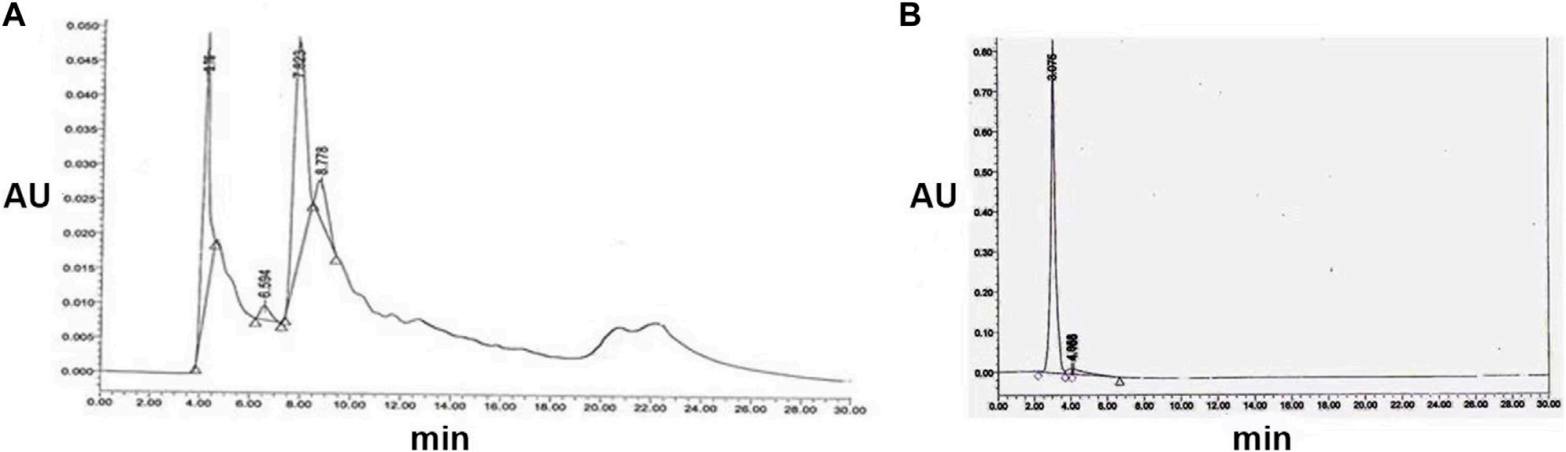
**(A)** Semi-preparative HPLC purification of antimicrobial compound from *S. albofaciens MS38* at 254 nm, showing different peaks at 4.11, 6.594, 7.823, and 8.778 min using water: acetonitrile (70:30) solvent system at 254 nm with a flow rate of 0.5 mL/min in a C-18 column **(B)** HPLC profile of purified compound by *S. albofaciens MS38*.

Chloroform (v/v) exhibited potent antibacterial and antifungal activity, with the zone of inhibition varying across silica column fractions containing methanol concentrations of 25%, 30%, 35%, 40%, 45%, 50%, and 55%. The antimicrobial fractions were consolidated and further purified via HPLC using a solvent system of water: acetonitrile (30:70, 45:55, 70:30, and 30:30 v/v) at a flow rate of 0.8 mL/min and detected at 254 nm. The initial antimicrobial fraction primarily consisted of two peaks with retention times of 3.76 and 7.823 min, along with an additional shoulder at 8.77 min (Figure 2A). Peak P1, with a retention time of 3.76 min, exhibited antibacterial and antifungal activities against *B. cereus* and *C. albicans*, resulting in inhibition zones of 25 mm and 23 mm, respectively. Subsequent purification of Peak P1 (3.79) involved HPLC employing a water: acetonitrile (70:30) solvent system at 254 nm with a flow rate of 0.5 mL/min in a C-18 column (Figure 2B). The final purified product, with a retention time of P1 at 3.075 min, showcased the highest antifungal and antibacterial activities.

### 3.6 Characterization of isolated compound

**FIGURE 3:**
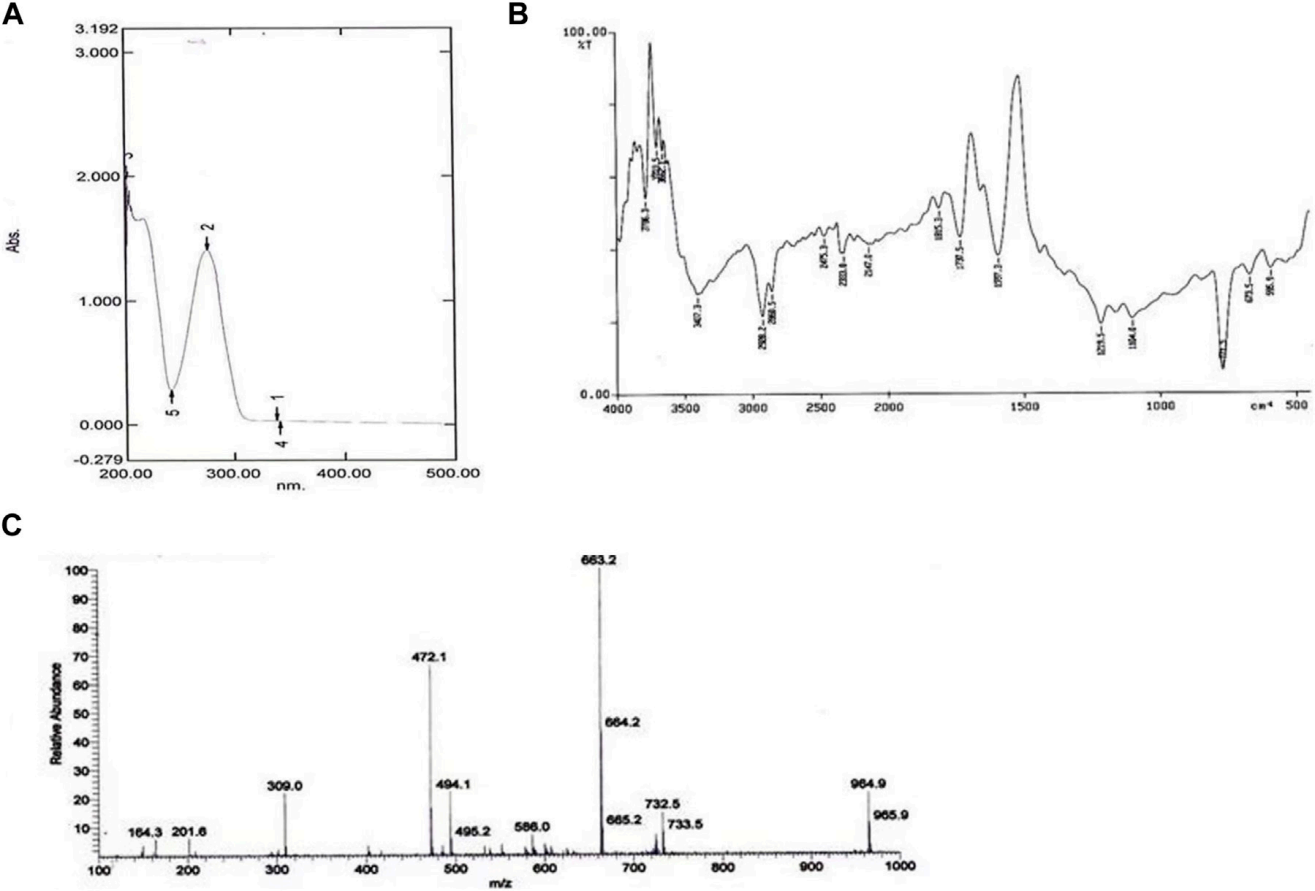
**(A)** UV spectra of purified compound by *S. albofaciens* MS38; **(B)** IR spectra of purified compound by *S. albofaciens* MS38; **(C)** Mass spectra (ESIMS) of purified compound produced by *S. albofaciens* MS38.

The UV absorbance spectrum in methanol (MeOH) provided a detailed insight into the structural features of the compound isolated from *S. albofaciens* MS38. Noteworthy peaks at 340 nm indicated the presence of pyrimidine, while a substantial peak at 275 nm suggested the existence of a carbonyl group (C=O). Additional peaks at 241 nm and 202 nm were attributed to the phenyl base peak and the presence of a furan ring, respectively (Figure 3A). These UV absorbance features contribute significantly to the elucidation of the compound’s electronic structure. The infrared (IR) spectrum, obtained using the KBr pellet technique, revealed distinctive peaks at specific wavenumbers, providing detailed information about the functional groups present. Peaks at 3786 cm^-1, 3704 cm^-1, and 3662 cm^-1 indicated the presence of hydroxyl groups (OH) and amino groups (NH and NH2), while the peak at 3407 cm^-1 suggested aromatic hydrogen (Ar-H). Other peaks provided insights into C-H stretching, carbonyl (C=O), and other chemical bonds (Figure 3B). Electrospray ionization mass spectrometry (ESIMS) analysis corroborated the molecular weight determination, with a base peak at m/z 472 (M+) aligning with the reported molecular weight of 471, confirming the presence of the isolated substance from S*. albofaciens* MS38 (Figure 3C). The presence of a single peak in mass spectra shows the purity of the compound purified from HPLC. Proton nuclear magnetic resonance (1HNMR) and carbon-13 nuclear magnetic resonance (13CNMR) analyses in D2O and CDCl3, respectively, provided detailed information on proton environments and carbon distributions within the compound (Supplementary Figures S1, S2). Elemental analysis conducted using the Carlo Erba 1106 CHNO/S elemental analyzer yielded calculated values of C = 56.04; H = 6.20; and N = 20.79, closely aligned with the observed values (Table 2). The refined product exhibited positive responses to ninhydrin, indicating the presence of amino groups, and negative responses to nitroprusside, tollen, molish, meyer, ferric chloride, Sakaguchi, lead sulfate, the Ehrlich reaction, and Millon’s reaction (Table 3). Collectively, these analyses offer a comprehensive understanding of the structural and chemical characteristics of the isolated compound from *S. albofaciens* MS38.

**TABLE 2:** A comparative study of the purified active compound by *S. albofaciens* MS38 with reference standard antibiotic.

Sr. No.	Chemical analysis	Purified antimicrobial agent	Puromycin* (standard)
1	C	56.04	56.04
2	H	6.20	6.20
3	N	20.79	20.79
4	Molecular weight	471	471
5	Melting point	167–170	165–172
6	Molecular Formula	^C^_22_^H^_29_^N^_7_^O^_5_	^C^_22_^H^_29_^N^_7_^O^_5_

**TABLE 3:** Chemical tests for the purified active compound produced by *S. albofaciens* MS38.

Sr. No.	Chemical test	Results	Remark
1	Tollen`s reaction	-ve	Aromatic aldehydes or ketones absent
2	Ninhydrin test	+ve	^Free−NH^_2_^group is present^
3	Millon`s reaction	-ve	Tyrosine is absent
4	Ehrlich reaction	-ve	No Indolic group
5	Meyer reaction	-ve	Nitro-group is absent
6	Lead sulphate reaction	-ve	Amino acids containing sulphur are absent
7	Sakagychi recation	-ve	Arginine is absent
8	Fehling`s reaction	+ve	Free aldhyde and/or keto sugars are present
9	Ferric chloride reaction	-ve	Di-ketons or enolic groups are absent
10	Nitroprusside reaction	-ve	Sulphur is absent
11	Molisch’s reaction	-ve	Sugar moiety is absent

### 3.7 Minimum inhibitory concentration (MIC) of isolated substance

The isolated substance from *S. albofaciens* MTCC 10793 displayed an impressive MICs profile (Table 4), showcasing broad-spectrum efficacy against both Gram-positive and Gram-negative bacteria, as well as fungi. Additionally, the purified substance demonstrated anti-tubercular activity against *Mycobacterium* TB H37Rv and H37Ra at concentrations of 6.25–12.5 g/mL. MIC values ranged from 3.125 to 6.25 g/mL against *S. aureus* (MTCC 96), *S. aureus* (MTCC 737), *Staphylococcus epidermis* (MTCC 435), *Salmonella typhi* (MTCC 531), *S. epidermis* (MTCC 435). 12.5–25 μg/mL against *Klebsiella pneumoniae* (MTCC 2405), *Proteus vulgaris* (MTCC 1771), *Bacillus megaterium* (MTCC 1684) and *Trichophyton rubrum* (MTCC 296), 12.5 μg/mL against *B. cereus* (MTCC 1305), *B. cereus* (ATCC 10876), *Escherichia coli* (ATCC 35218), *Candida tropicalis* (MTCC 184), *C. tropicalis* (MTCC 3017), *C. albicans* (MTCC 183) *C. albicans* (MTCC 184), *T. rubrum* (JNMC), *Aspergillus niger* (MTCC 872) *Aspergillus fumigatus* (MTCC 2544), 6.25–12.5 μg/mL against *Bacillus subtilis* (JNMC), *B. subtilis* (MTCC 1789), *E. coli* (MTCC 1667), *E. coli* (MTCC 739), *S. aureus* (JNMC), *S. typhi* (JNMC), *C. albicans* (MTCC 1637), *C. albicans* (JNMC), *Alterneria alternate* (MTCC 1779) *Sachromyces cereviseae* (MTCC170), *Penicillium citrinum* (MTCC 1751), 25–50 μg/mL against *Cryptococcus terreus* (MTCC 1716), *Bacillus pumilus* (MTCC 1607). In conclusion, this study presents a thorough investigation of a novel broad-spectrum antibacterial strain isolated from Chhattisgarh, India. The strain, identified as *S. albofaciens*, was found to produce an antimicrobial compound with significant potential for pharmaceutical applications, including its notable efficacy against various pathogenic organisms. The elucidation of its molecular structure and comprehensive characterization further contribute to its potential as a valuable bioactive agent.

**TABLE 4:** The minimum inhibitory concentration of the purified compound of *S. albiofaciens* MS38.

Sr. No.	Test pathogens used	Purified compound (µg/mL)
1	*Mycobacterium tuberculosis* H37*Rv*	6.25–12.5
2	*Mycobacterium tuberculosis* H37*Ra*	6.25–12.5
3	*Bacillus subtilis* (MTCC 1789)	6.25–12.5
4	*Bacillus subtilis* (JNMC)	6.25–12.5
5	*Bacillus pumilus* (MTCC 1607)	25–12.5
6	*Klebsiella pneumoniae* (MTCC 2405)	12.5–25
7	*Escherichia coli* (MTCC 1667)	6.25–12.5
8	*Staphylococcus aureus* (MTCC 96)	3.125–6.25
9	*Staphylococcus aureus* (JNMC)	6.25–12.5
10	*Staphylococcus aureus* (MTCC 737)	3.125–6.25
11	*Staphylococcus epidermis* (MTCC 435)	3.125–6.25
12	*Proteus vulgaris* (MTCC 1771)	12.5–25
13	*Salmonella typhi* (MTCC 531)	3.125–6.25
14	*Salmonella typhi* (JNMC)	6.25–12.5
15	*Bacillus megaterium* (MTCC 1684)	12.5–25
16	*Bacillus cereus* (MTCC 1305)	12.5
17	*Bacillus cereus* (ATCC 10876)	12.5
18	*Escherichia coli* (ATCC 35218)	12.5
19	*Escherichia coli* (MTCC 739)	6.25–12.5
20	*Candida tropicalis* (MTCC 184)	12.5
21	*Candida tropicalis* (MTCC 3017)	12.5
22	*Candida albicans* (JNMC)	6.25–12.5
23	*Candida albicans* (MTCC 1637)	6.25–12.5
24	*Candida albicans* (MTCC 183)	12.5
25	*Candida albicans* (MTCC 184)	12.5
26	*Cryptococcus terreus* (MTCC 1716)	25–50
27	*Trichophyton rubrum* (MTCC 296)	12.5–25
28	*Trichophyton rubrum* (JNMC)	12.5
29	*Aspergillus niger* (MTCC 872)	12.5
30	*Aspergillus fumigatus* (MTCC 2544)	12.5
31	*Alterneria alternate* (MTCC 1779)	6.25–12.5
32	*Sachromyces cereviseae* (MTCC170)	6.25–12.5
33	*Penicillium citrinum* (MTCC 1751)	6.25–12.5

## 4 Discussion and conclusion

The quest for novel antibiotics and bioactive microbial compounds has gained immense importance due to the pressing challenge of bacterial antibiotic resistance (Miethke et al., 2021). In response to the escalating need for fresh antibiotics, our study focuses on isolating a potent antimicrobial strain from the soils of Chhattisgarh. This strain demonstrates a remarkable ability to combat a wide spectrum of pathogenic and nonpathogenic organisms, as well as exhibit promising anticancer properties. Identification of the strain as *S. albofaciens* was based on a comprehensive assessment of its morphological, cultural, physiological, and biochemical attributes, complemented by a thorough analysis of the 16S rRNA gene. Employing liquid fermentation within a glucose-soybean medium, we achieved substantial production of a potent antibacterial compound. Notably, our investigation unveiled that maximal production of the active metabolite occurs during the late log phase, revealing an inverse correlation between growth rate and metabolite yield. This phenomenon has parallels with instances of antibiotic accumulation such as cephalomycin C and clavulanic acid (Perry et al., 2022). Furthermore, the presence of a fast-metabolizing compound like glucose has been shown to enhance the production of antifungal metabolites through catabolite suppression (Singh et al., 2012). Soybean meal, known to be an optimal organic nitrogen source, played a pivotal role in our approach. Its rich amino acid composition, encompassing lysine, methionine, tryptophan, and more, significantly stimulates antibiotic synthesis, in line with prior studies (Singh et al., 2009). To achieve a higher degree of purity, the partially purified compound obtained from the silica column was subjected to reverse-phase HPLC (Satyajit et al., 2015). Subsequent structural analyses using UV, IR, MS, and NMR unequivocally identified the primary antimicrobial compound as puromycin. This achievement bears unique significance, as our strain marks the initial producer of the antibiotic puromycin. This compound acknowledged for its anticancer properties and role as a protein synthesis inhibitor, was previously attributed to *S. alboniger* (Niu et al., 2015). Remarkably, our study spotlights the potential of *Streptomyces* from diverse taxonomic groups in yielding novel primary and secondary metabolites, further underscoring the untapped reservoir of microbial natural products. Yet, the understanding of *Streptomyces* sp. and its prolific potential remains comparatively limited. Our findings strongly validate the hypothesis that novel actinomycete species offer a rich source of advantageous secondary metabolites. In a landscape where existing medications often possess limited efficacy and adverse effects, the emergence of novel antibacterial compounds from natural origins offers promising alternatives for disease treatment (Ye et al., 2020). Natural substances can bring forth reduced risks of adverse effects and economic advantages. The surge of interest in harnessing natural resources for combating serious diseases is gaining momentum. Our comprehensive investigation delved into the microbial diversity of Chhattisgarh, revealing the potent antimicrobial capabilities of the MS38 isolate identified as *S. albofaciens*. This pioneering work culminated in the discovery of puromycin as a pharmaceutically active compound, underlining the potential of this strain for future therapeutic applications. Additionally, the scale-up of production processes and the optimization of fermentation conditions are essential for practical applications.

## Data Availability

The datasets presented in this study can be found in online repositories. The names of the repository/repositories and accession number(s) can be found in the article/Supplementary Material.

## References

[B1] Alam K. Mazumder A. Sikdar S. Zhao Y. M. Hao J. Song C. (2022). Streptomyces: the biofactory of secondary metabolites. Front. Microbiol. 13, 968053. 10.3389/fmicb.2022.968053 36246257 PMC9558229

[B2] Atanasov A. G. Zotchev S. B. Dirsch V. M. (2021). International natural product Sciences taskforce. Nat. Rev. Drug Discov. 20 (3), 200–216. 10.1038/s41573-020-00114-z 33510482 PMC7841765

[B3] Aviner R. (2020). The science of puromycin: from studies of ribosome function to applications in biotechnology. Comput. Struct. Biotechnol. J. 18, 1074–1083. 10.1016/j.csbj.2020.04.014 32435426 PMC7229235

[B4] Balouiri M. Sadiki M. Ibnsouda S. K. (2016). Methods for *in vitro* evaluating antimicrobial activity: a review. J. Pharm. analysis 6 (2), 71–79. 10.1016/j.jpha.2015.11.005 PMC5762448 29403965

[B5] Caicedo-Montoya C. Manzo-Ruiz M. Ríos-Estepa R. (2021). Pan-Genome of the genus Streptomyces and prioritization of biosynthetic gene clusters with potential to produce antibiotic compounds. Front. Microbiol. 12, 677558. 10.3389/fmicb.2021.677558 34659136 PMC8510958

[B6] Chiu S. Wang T. Belski M. Abourashed E. A. (2016). HPLC-guided isolation, purification and characterization of phenylpropanoid and phenolic constituents of nutmeg kernel (myristica fragrans). Nat. Product. Commun. 11 (4), 483-488. 10.1177/1934578X1601100416 PMC4943679 27396199

[B7] Clsi (2020). Performance standtard for antimicrobial testing. 30th. Wyane PA, USA: Clinical and Laboratory Standards Institute.

[B8] EclinicalMedicine (2021). Antimicrobial resistance: a top ten global public health threat. EClinicalMedicine 41, 101221. 10.1016/j.eclinm.2021.101221 34877512 PMC8633964

[B9] Gomez-Escribano J. P. Bibb M. J. (2014). Heterologous expression of natural product biosynthetic gene clusters in Streptomyces coelicolor: from genome mining to manipulation of biosynthetic pathways. J. industrial Microbiol. Biotechnol. 41 (2), 425–431. 10.1007/s10295-013-1348-5 24096958

[B10] Liu A. W. Villar-Briones A. Luscombe N. M. Plessy C. (2022). Automated phenol-chloroform extraction of high molecular weight genomic DNA for use in long-read single-molecule sequencing. F1000Research 11, 240. 10.12688/f1000research.109251.1 35350547 PMC8931447

[B11] Mantravadi P. K. Kalesh K. A. Dobson R. C. J. Hudson A. O. Parthasarathy A. (2019). The quest for novel antimicrobial compounds: emerging trends in research, development, and technologies. Antibiot. (Basel, Switz. 8 (1), 8. 10.3390/antibiotics8010008 PMC6466574 30682820

[B12] Miethke M. Pieroni M. Weber T. Brönstrup M. Hammann P. Halby L. (2021). Towards the sustainable discovery and development of new antibiotics. Nat. Rev. Chem. 5 (10), 726–749. 10.1038/s41570-021-00313-1 PMC8374425 34426795

[B13] Niu G. Tan H. (2015). Nucleoside antibiotics: biosynthesis, regulation, and biotechnology. Trends Microbiol. 23 (2), 110–119. 10.1016/j.tim.2014.10.007 25468791

[B14] Pancu D. F. Scurtu A. Macasoi I. G. Marti D. Mioc M. Soica C. (2021). Antibiotics: conventional therapy and natural compounds with antibacterial activity-A pharmaco-toxicological screening. Antibiot. (Basel, Switz. 10 (4), 401. 10.3390/antibiotics10040401 PMC8067816 33917092

[B15] Perry E. K. Meirelles L. A. Newman D. K. (2022). From the soil to the clinic: the impact of microbial secondary metabolites on antibiotic tolerance and resistance. Nat. Rev. Microbiol. 20 (3), 129–142. 10.1038/s41579-021-00620-w 34531577 PMC8857043

[B16] Procópio R. E. Silva I. R. Martins M. K. Azevedo J. L. Araújo J. M. (2012). Antibiotics produced by Streptomyces. Braz J. Infect. Dis. 16 (5), 466–471. 10.1016/j.bjid.2012.08.014 22975171

[B17] Satyajit D. Lutfun N. (2015). Applications of high performance liquid chromatography in the analysis of herbal products. Evidence-Based Validation Herb. Med., 405–425. 10.1016/B978-0-12-800874-4.00019-2

[B18] Serwecińska L. (2020). Antimicrobials and antibiotic-resistant bacteria: a risk to the environment and to public health. Water 12, 3313. 10.3390/w12123313

[B19] Singh N. Rai V. (2013). *In vitro* antimycotic activity of a new isolate Streptomyces fradiae MTCC 11051 against the multi-drug resistant pathogenic fungi. J. Pharm. Res. 7 (4), 331–336. 10.1016/j.jopr.2013.04.024

[B20] Singh N. Rai V. Tripathi C. (2012). Production and optimization of oxytetracycline by a new isolate Streptomyces rimosus using response surface methodology. Med. Chem. Res. 21 (10), 3140–3145. 10.1007/s00044-011-9845-4

[B21] Singh N. Rai V. Tripathi C. K. (2013). Purification and chemical characterization of antimicrobial compounds from a new soil isolate Streptomyces rimosus MTCC 10792. Prikl. biokhimiia i Mikrobiol. 49 (5), 473–480. 10.1134/s0003683813050116 25474869

[B22] Singh V. Khan M. Khan S. Tripathi C. K. (2009). Optimization of actinomycin V production by Streptomyces triostinicus using artificial neural network and genetic algorithm. Appl. Microbiol. Biotechnol. 82 (2), 379–385. 10.1007/s00253-008-1828-0 19137288

[B23] Umezawa H. (1977). Recent advances in bioactive microbial secondary metabolites. Jpn. J. antibiotics 30, 138–163. 77338

[B24] Wang Q. Q. Li M. X. Li C. Gu X. X. Zheng M. Z. Chen L. X. (2020). Natural products and derivatives targeting at cancer energy metabolism: a potential treatment strategy. Curr. Med. Sci. 40 (2), 205–217. 10.1007/s11596-020-2165-5 32337682

[B25] Who (2021). AWaRe classification. Geneva, Switzerland: WHO.

[B26] Ye L. Zhang J. Xiao W. Liu S. (2020). Efficacy and mechanism of actions of natural antimicrobial drugs. Pharmacol. Ther. 216, 107671. 10.1016/j.pharmthera.2020.107671 32916205

