# Supplementary material for: Green extraction of puromycin-based antibiotics from Streptomyces albofaciens (MS38) for sustainable biopharmaceutical applications
Source: Front Chem. 2024 Jan 9;11:1326328. doi: 10.3389/fchem.2023.1326328 (PMC10803528; doi:10.3389/fchem.2023.1326328)
Supplement: Supplementary file 1 [file DataSheet1.docx]

**Supplementary figures**

**Green Extraction of Puromycin-Based Antibiotics from *Streptomyces albofaciens* (MS38) for Sustainable Biopharmaceutical Applications**

**Neha Singh^1,2†,^ Sandip Patil^3,4†^, Mohd. Shahnawaz^5^, Vibhuti Rai^1^, Abhinandan Patil^6^ CKM Tripathi^7^, Feiqiu Wen^3^, Shaowei Dong^3#^ and** **Defeng Cai^8#^**

1. Biochemistry and Microbiology Laboratory, School of Studies in Life Sciences, Pt. Ravishsankar Shukla University, Raipur, 492010, India
2. Virology Lab, Department of Microbiology, Pandit Jawahar Lal Nehru Memorial Medical College, Raipur, Chhattisgarh, 492001, India
3. Department of Haematology and Oncology, Shenzhen Children’s Hospital, Shenzhen, Guangdong Province, 518038 China
4. Paediatric Research Institute, Shenzhen Children’s Hospital, Shenzhen, Guangdong Province, 518038 China
5. Department of Botany, University of Ladakh, Kargil Campus, Khumbathang-194105, Ladakh UT, India
6. Division of Pharmacy, Dr DY Patil University, Kolhapur, Maharashtra, 416006 India
7. Fermentation Technology Division, Central Drug Research Institute, CSIR, Chattar Manzil Palace, PO Box 173, Lucknow 226 001, India
8. Clinical Laboratory (Pathology) Centre, South China Hospital of Shenzhen University, Shenzhen, Guangdong Province, 518038 China

**†** These authors share first authorship

**#Correspondence:**

**Shaowei Dong:** michael.dong.85@gmail.com (S.D)

**Defeng Cai:** caidefeng0755@126.com (D.C.)

**Key Words**: *Streptomyces albofaciens strain MS38*, Secondary metabolites, antimicrobial activity, Puromycin and Bioactive compound

**Running Title:** Novel *S. albofaciens* (MS38) Producing Puromycin-Based Antibiotics


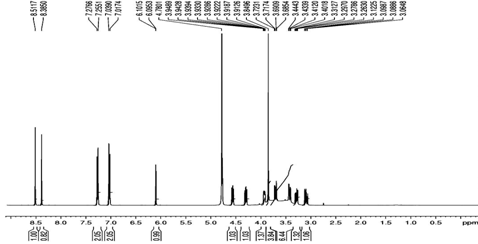


Fig. S1. 1H NMR spectra of the purified compound from *S. albofaciens* MS38


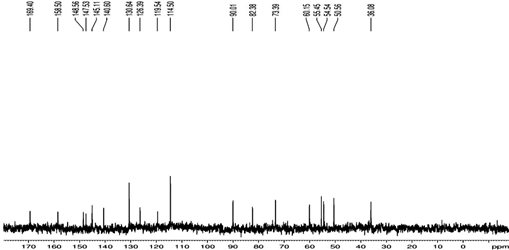


Fig. S2. C13 NMR spectra of the purified compound by *S. albofaciens* MS38

Table S1: List of tested microorganisms

*Mycobacterium tuberculosis* H37Rv, *Mycobacterium tuberculosis* H37Ra, *Bacillus subtilis* (MTCC 1789), *Bacillus subtilis* (JNMC), *Bacillus pumilus* (MTCC 1607), *Bacillus megaterium* (MTCC 1684), *Bacillus cereus* (MTCC 1305), *Bacillus cereus* (ATCC 10876), *Staphylococcus aureus* (MTCC 96), *Staphylococcus aureus* (JNMC), *Staphylococcus aureus* (MTCC 737), *Staphylococcus epidermis* (MTCC 435), *Salmonella typhi* (MTCC 531), *Salmonella typhi* (JNMC), *Proteus vulgaris* (MTCC 1771),  *Klebsiella pneumoniae* (MTCC 2405), *Escherichia coli* (MTCC 1667), *Escherichia coli* (MTCC 739), *Escherichia coli* (MTCC 1687), *Escherichia coli* (ATCC 35218), *Candida albicans* (MTCC 1637), *Candida albicans* (JNMC), *Candida albicans* (MTCC 184), *Candida albicans* (MTCC 183), *Candida tropicalis* (MTCC 3017), *Aspergillus niger* (MTCC 872),  *Aspergillus fumigatus* (MTCC 2544), *Alterneria alternate*  (MTCC 1779),  *Penicillium citrinum*  (MTCC 1751), *Sachromyces cereviseae* (MTCC 170), *Tricophyton rubrum* (JNMC), *Tricophyton rubrum* (MTCC 296).
